# Expression of MICA, MICB and NKG2D in human leukemic myelomonocytic and cervical cancer cells

**DOI:** 10.1186/1756-9966-30-37

**Published:** 2011-04-10

**Authors:** Benny Weiss-Steider, Isabel Soto-Cruz, Christian A Martinez-Campos, Jorge Flavio Mendoza-Rincon

**Affiliations:** 1 Laboratorio de Oncología Molecular. Unidad de Diferenciación Celular y Cáncer. FES-Zaragoza, Universidad Nacional Autónoma de México. Ciudad de México. 09230. Mexico

## Abstract

**Background:**

Cancer cells are known to secrete the stress molecules MICA and MICB that activate cytotoxicity by lymphocytes and NK cells through their NKG2D receptor as a mechanism of immunological defense. This work was undertaken to evaluate if cancer cells can also express this receptor as a possible mechanisms of depletion of MIC molecules and thus interfere with their immune recognition.

**Methods:**

Myelomonocytic leukemic (TPH-1 and U-937) and cervical cancer (CALO and INBL) cell lines were evaluated by Western Blot, ELISA, flow cytometry and immunocytochemistry to evaluate their capacity to express and secrete MICA and MICB and to be induced to proliferate by these molecules as well as to express their receptor NKG2D. Statistical analysis was performed by two-way ANOVA for time course analysis and Student's t-test for comparison between groups. Values were considered significantly different if p < 0.05.

**Results:**

THP-1 and U-937 produce and secrete the stress MICA and MICB as shown by Western Blot of lysed cells and by ELISA of their conditioned media. By Western Blot and flow cytometry we found that these cells also express the receptor NKG2D. When THP-1 and U-937 were cultured with recombinant MICA and MICB they exhibited a dose dependent induction for their proliferation. CALO and INBL also produce MICA and MICB and were induced to proliferate by these stress molecules. By Western Blot, flow cytometry and immunocytochemistry we also found that these cells express NKG2D.

**Conclusions:**

Our novel results that tumor cells can simultaneously secrete MIC molecules and express their receptor, and to be induced for proliferation by these stress molecules, and that tumor epithelial cells can also express the NKG2D receptor that was thought to be exclusive of NK and cytotoxic lymphocytes is discussed as a possible mechanism of immunological escape and of tumor growth induction.

## Background

NKG2D is a member of the NKG2 family of HLA class I C-type lectin receptors and is expressed as a homodimer by NK cells [1,2] and cytotoxic lymphocytes [3,4]. The ligands for NKG2D include the human class I-like molecules MICA and MICB [5], which are stress-induced molecules expressed by tumors of epithelial origin [6,7] and, leukemias [8], as well as by virus-infected cells [9,10]. The recognition of the MICA and MICB ligands on tumor cells by the NKG2D receptor, found on NK cells, induces the cytotoxic activity of NK cells [11] and the subsequent lysis of their tumor targets [12]. The secretion of MICA and MICB by cancer cells has been suggested as a mechanism for tumor cell immune escape through the saturation of NKG2D receptors on cytotoxic cells [13,14], thus abrogating their ability to recognize tumor cells. In fact, high levels of these molecules were found in the sera of human cancer patients [15], and a direct correlation was found between increased serum concentrations of these molecules and tumor stage [16].

It is not known if the secretion of MICA and MICB by the tumor cells has any effect on the cancer cells themselves. This work was undertaken to determine if two human leukemic myelomonocytic cell lines, THP-1 and U-937, produce MICA and MICB and express NKG2D, and if these stress molecules induce cell proliferation. In order to determine if these properties are shared by other tumors, we also analyzed the CALO and INBL human epithelial cervical cancer cell lines.

## Methods

### Cells and antibodies

The U-937 and THP-1 cell lines were purchased from ATCC (American Type Culture Collection), whereas CALO and INBL were established in our laboratory [17,18]. The cells were cultured at 37°C with 5% CO_2 _in RPMI-1640 medium (Invitrogen) supplemented with 10% heat-inactivated FCS (Hyclone), 1-mM MEM sodium pyruvate solution, 2-mM MEM non-essential amino acids solution (Gibco), 0.1-mM L-glutamine, 100-U/ml penicillin and 100-μg/ml streptomycin (Gibco). Polyclonal antibody against MICA/MICB and murine monoclonal anti-MICA, anti-MICB and anti-NKG2D antibodies were purchased from R&D Systems.

### Proliferation assays

U-937 and THP-1, as well as CALO and INBL, cells were plated at 5 × 10^3 ^cells per well in 96-well plates. Cells were treated with different concentrations of either MICA or MICB for 72 h at 37°C with 5% CO_2 _in RPMI-1640 containing 10% FCS. Proliferation was measured using the MTT assay (3-[4,5-Dimethylthiazol-2-4]-2,5-diphanyltetrazolium bromide) (Sigma). Briefly, 5 × 10^3 ^cells were cultured for 72 h in the presence of 1, 10, or 100 ng recombinant human MICA or MICB protein. MTT reagent was then added and the plates were read in a micro-titer plate reader at 570 nm.

### Cell lysis and immunoblotting

For immunoprecipitation, 10^7 ^cells were lysed for 15 min at 4°C in a lysis buffer (50-mM Tris-HCl, pH 7.4, 150-mM NaCl, 5-mM EDTA, 10-mM NaF, 1-mM sodium orthovanadate, 1-mM phenylmethanesulfonyl fluoride, 1-μg/ml leupeptin, 1-μg/ml pepstatin, 1-μg/ml aprotinin and 1% Triton X-100). The insoluble material was pelleted (15,000 × *g *for 15 min) at 4°C.

Total protein content in the lysates was determined using the Bio-Rad protein assay (Bio-Rad), and 150 μg of protein was incubated with protein A-agarose beads (Invitrogen) previously coupled with the corresponding antibody. The immune complexes were washed five times with cold washing buffer (50-mM Tris-HCl, pH 7.4, 150-mM NaCl, 5-mM EDTA, 10-mM NaF, 1-mM sodium orthovanadate, 1-mM phenylmethanesulfonyl fluoride, 1-μg/ml leupeptin, 1-μg/ml pepstatin, 1-μg/ml aprotinin and 0.1% Triton X-100) and resolved by SDS-PAGE (10% acylamide).

To obtain total cell lysates, 10^7 ^cells were washed once with ice-cold phosphate-buffered saline (PBS) in a microfuge tube. Pellets were rapidly resuspended in 40 μL of lysis buffer, incubated for 15 min on ice and insoluble material was pelleted (15,000 × *g *for 15 min) at 4°C. Forty microliters of 2× Laemmli sample buffer (120-mM/L Tris, pH 6.8, 2-mM urea, 100-mM/L DTT, 10% glycerol and 0.001% bromophenol blue) were immediately added while vortexing, and the sample was boiled for 5 min. Fifty microliters of each sample, along with molecular weight markers (Bio-Rad), were electrophoresed by vertical SDS-PAGE.

The proteins were electroblotted onto nitrocellulose membranes, and the membranes were blocked overnight in TBST buffer (10-mM Tris-HCl, pH 7.4, 100-mM NaCl and 0.5% Tween 20) containing 3% BSA. For protein immunodetection, the membranes were subjected to immunoblotting with 1 μg/ml of the appropriate antibody for 1.5 h at room temperature followed by HRP-conjugated anti-mouse or anti-rabbit IgG diluted to 1:6,000 (Zymed) for 30 min at room temperature. The membranes were then washed five times in TBST and the bands were visualized using the ECL system, according to the manufacturer's instructions (Pierce).

### ELISA assay

For ELISA assays, 5 × 10^4 ^U-937 and THP-1, as well as CALO and INBL, cells were plated in 48-well plates for 7 days. The cell culture supernatants were collected every 24 h and stored at -70°C until use, and ELISA detection was performed using 100 μL of each supernatant. In brief, plates were coated with 100 μL of the supernatants from the leukemic myelomonocytic and cervical cancer cells by incubating at 37°C for 1 h, washing three times with PBS-Tween (PBST) and blocking with 120 μL of PBST-3% BSA for 1 h at 37°C. Monoclonal antibodies (1:100 in PBST-3% BSA) were added for 1 h at 37°C. Anti-mouse IgG2a-HRP (1:4000 in PBST-3% BSA) was added for 1 h at 37°C. Plates were then washed and developed using 100 μL of ABTS system substrate (Zymed). The absorbance was measured at 405 nm.

### Immunohistochemical analysis of NKG2D

Immunohistochemical staining for the expression of NKG2D was completed by standard procedures. In brief, CALO and INBL cell lines were seeded onto poly-L-lysine-coated microscopy slides and allowed to grow for 72 h. Cells were heated in citrate buffer (0.01 mol/L, pH 6.0) in a microwave oven (85-95°C, 3 times for 5 min each) followed by blocking the nonspecific binding sites with goat serum. Cells were incubated with the primary mouse monoclonal anti-NKG2D antibody (R&D Systems) overnight in a humidified chamber at 4°C. The samples were then incubated with a polyclonal goat anti-rabbit HRP-conjugated secondary antibody for 30 min at room temperature. Slides were then processed with the universal LSAB-2 single reagents (peroxidase) kit, and the expression of NKG2D was identified by enzyme development with diaminobenzidine. As a final step, the slides were stained with methylene blue counterstaining and dehydrated in graded alcohols. Negative control slides were processed similarly, except with the primary antibody omitted, and incubated with an irrelevant isotype antibody. Immunohistochemical staining was examined using a light microscope (Leica D100) equipped with a digital camera.

### Expression of surface NKG2D by flow cytometry

Cell suspensions (0.4 × 10^6 ^cells/ml) in PBS with 5% FBS and 0.01% azide were incubated with 10 μg/ml of the primary murine monoclonal anti-NKG2D antibody or the respective isotype control for 90 min at 4°C. After washing the cells with PBS, they were incubated in the dark for 30 min with 0.45-μg/ml FITC-labeled goat anti-mouse IgG at 4°C. After washing again, the cells were fixed for 20 min in 1% paraformaldehyde, followed by two more washes. The stained cells were analyzed in a FACScan cytometer (Becton Dickinson).

### Isolation of human monocytes

Human monocytes were isolated from peripheral blood samples of healthy donors by Ficoll-Paque density gradient centrifugation and plastic adherence purification. Cell viability was greater than 95%, as assessed by trypan blue exclusion, and the purity of monocytes was greater than 93%, as determined by immunofluorescent staining with anti-CD14 monoclonal antibody (Becton Dickinson) and flow cytometric analysis.

### Statistical analysis

All data are expressed as the mean ± SD of three replicates, and all experiments were repeated three times, unless otherwise stated. Statistical analysis was performed by two-way ANOVA for the time course analysis and Student's t-test for the comparison between groups. Values were considered significantly different if p < 0.05.

All reagents were from Sigma Chemical Co., San Louis, MO, USA, unless otherwise specified.

## Results

### The leukemic myelomonocytic U-937 and THP-1 cell lines produce and secrete MICA and MICB

**Figure 1:**
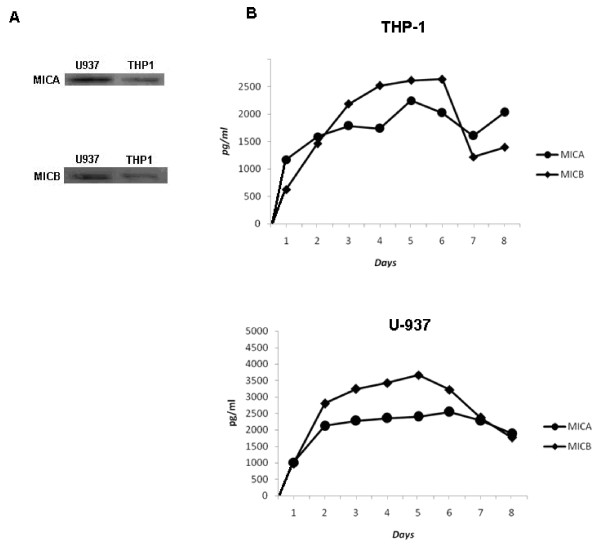
**Leukemic myelomonocitic cells express and secrete MICA and MICB**. THP-1 and U937 cells (1 × 10^7^) were lysed, proteins were immunoprecipitated and equal amounts of proteins from the total lysates were resolved by SDS-PAGE and transferred to nitrocellulose membranes. The blot was developed using either anti-MICA monoclonal antibodies or anti-MICB monoclonal antibodies (A) and an appropriate secondary antibody conjugated to HRP for chemiluminescent detection. THP-1 and U937 cells (50 × 10^3^) were cultured in 48-well plates for 7 days, and the conditioned media were collected daily. MIC proteins were detected by ELISA assay using specific antibodies. The production of MICA and MICB was evaluated using monoclonal antibodies against MICA and MICB in THP-1 and U-937 cells (B). Standard deviations were less than 5%

In order to evaluate if the leukemic myelomonocytic U-937 and TPH-1 cell lines produce MICA and MICB, we performed a western blot analysis using specific antibodies against MICA and MICB and found that both proteins were expressed in both cell lines (Figure 1A). To determine if the cells secreted MICA and MICB, we cultivated 5 × 10^3 ^cells for up to eight days and evaluated the amounts of these proteins in their respective conditioned media (CM). Using ELISA, we determined that MICA and MICB were indeed secreted into the CM from the first day of culture (Figure 1B). We did not find any MICA or MICB in the conditioned media of normal monocytes that were cultured under the same conditions as the myelomonocytic cells.

### U-937 and THP-1 proliferate in response to MICA and MICB

**Figure 2:**
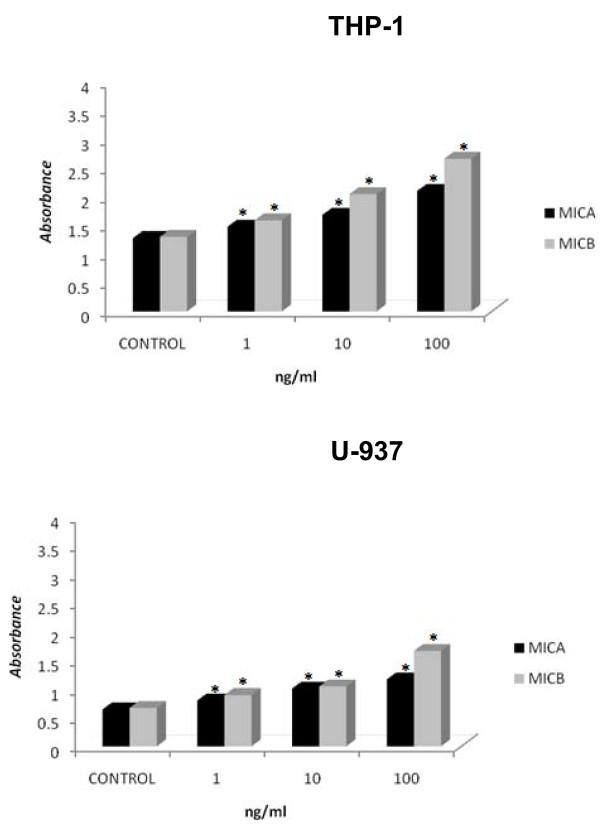
**MICA and MICB induce leukemic myelomonocytic cell line proliferation**. TPH-1 and U937 cells (5 × 10^3^) were cultured for 72 h in 96-well plates in the presence of 1, 10, or 100 ng recombinant human MICA or MICB. Proliferation was assayed using the MTT technique. The evaluation of THP-1 (A) and U-937 (B) cell proliferation. * indicates p < 0.05

After we detected that MICA and MICB were secreted by U-937 and THP-1 cells, we determined if external MICA and MICB could modulate their proliferation. For this purpose, we cultured 5 × 10^3 ^U-937 and TPH-1 cells for 3 days in the presence of 1, 10, or 100 ng of MICA or MICB and observed that both proteins induced significant dose-dependent proliferation (Figure 2). Normal monocytes were cultured in the same conditions as the myelomonocytic cells and no proliferation was obtained.

### U-937 and TPH-1 express NKG2D

**Figure 3:**
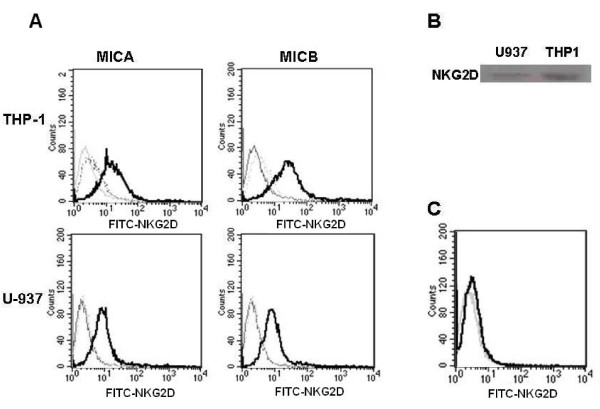
**NKG2D is expressed in leukemic myelomonocytic cell lines**. Flow cytometric analysis of NKG2D expression in the leukemic myelomonocytic TPH-1 and U937 cell lines in the presence of either MICA or MICB (A) and in normal blood monocytes under the same conditions (C). NKG2D was also detected by western blot analysis in THP-1 and U937 cells (B). The NKG2D levels in the isotype controls (dotted lines), non-treated cells (grey line) and MIC-treated cells with either 10 ng MICA or MICB for 18 h (solid lines) are depicted in the graphs.

After we demonstrated that the leukemic myelomonocytic cell lines proliferated in response to exogenous MICA and MICB, we evaluated the possible expression of NKG2D, which is the specific receptor for these proteins. Flow cytometry (Figure 3A) and western blot analysis (Figure 3B) using specific antibody against this receptor were used to show that U-937 and THP-1 cells do express NKG2D. Monocytes were used in the cytometry assay as a negative control (Figure 3C). It is interesting to note that we could only detect NKG2D by flow cytometry when the cells were previously activated for 18 h by either MICA or MICB.

### The CALO and INBL cervical cancer cell lines secrete MICA and MICB and express NKG2D

**Figure 4:**
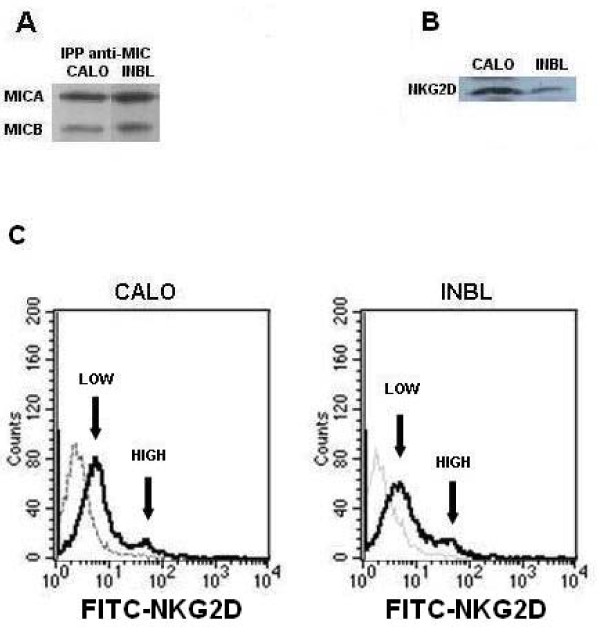
**Cervical cancer cell lines express MICA, MICB and NKG2D**. CALO and INBL cells (**1 × 10**^**7**^) were lysed proteins immunoprecipitated and equal amounts of protein from total lysates were resolved by SDS-PAGE and transferred to nitrocellulose membranes. The blots were developed with either polyclonal anti-MIC antibodies (A) or monoclonal anti-NKG2D antibodies (B) and an appropriate secondary antibody conjugated to HRP for chemiluminescence detection. Flow cytometric analysis of NKG2D expression in cervical carcinoma cell lines after 72 h induction with 10 ng MICB (C). We used only MICB to induce the expression of NKG2D because we previously obtained that MICB was a better inducer of myelomonocytic cell proliferation than MICA. Graphs show NKG2D levels (solid line) and isotype controls (dotted line).

**Figure 5:**
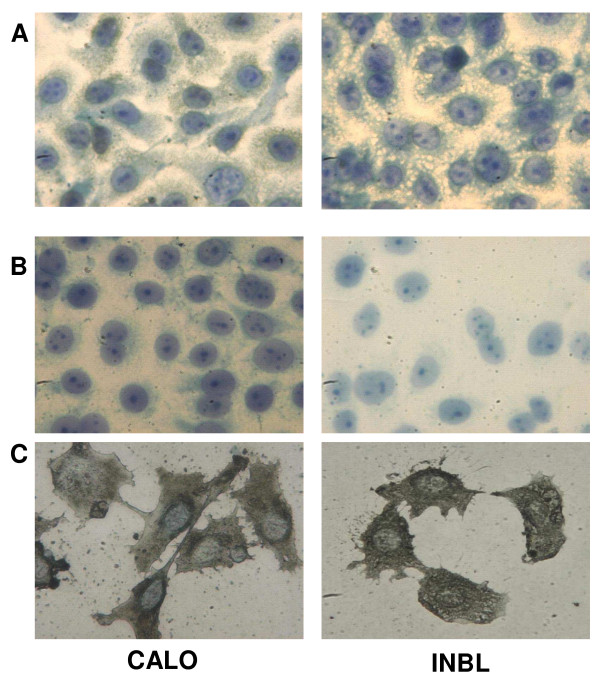
**Immunohistochemical localization of NKG2D in cervical cancer cell lines**. Adherent cells were preincubated with 10 ng of MICB for 72 h and then incubated with an anti-NKG2D primary antibody followed by an HRP-conjugated secondary antibody, and the samples were developed with diaminobenzidine and counterstained with methylene blue. Negative control (A), isotype control (B) and NKG2D staining (C) of CALO (left panels) and INBL (right panels) cells. Note the strong cytoplasmic staining in both cell lines. (Original magnification × 40)

**Figure 6:**
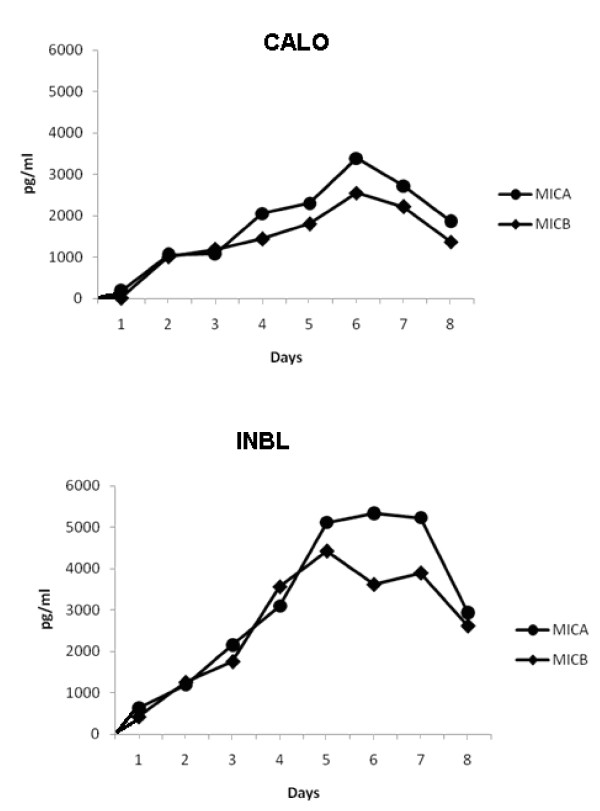
**Cervical cancer cell lines secrete MICA and MICB**. Cells (5 × 10^3^) were cultured in 48-well plates for 7 days, the supernatants were collected every 24 h, and MICA and MICB proteins were detected by ELISA using specific monoclonal antibodies. Data from CALO (A) and INBL (B) cells are shown.

In order to evaluate the capacity of other tumor cell types to express MICA and MICB, as well as NKG2D, we evaluated the possible expression of these proteins in two human epithelial cervical cancer cell lines, CALO and INBL, using polyclonal antibodies against MICA/MICB and anti-NKG2D for western blot and flow cytometric analyses. Our results show that MICA, MICB and NKG2D were expressed in both cell lines (Figs. 4A and 4B). It is interesting to mention that when flow cytometric analysis for NKG2D expression was performed after the cells were activated for 72 h by MICB, only a small minority of the cells exhibited high NKG2D expression, while the majority of the cells expressed low levels of the receptor (Figure 4C). The presence of NKG2D was further evaluated by immunohistochemical analysis, which revealed a reproducible pattern of staining in both cervical cancer cell lines (Figure 5). We also evaluated if CALO and INBL secreted MICA and MICB into their culture media. For this purpose, we seeded 5 × 10^3 ^cells for up to eight days and detected significant amounts of MICA and MICB in the CM by ELISA; the concentration of MICA AND MICB increased during the first five days in culture (Figure 6).

### CALO and INBL proliferate in response to MICA and MICB

**Figure 7:**
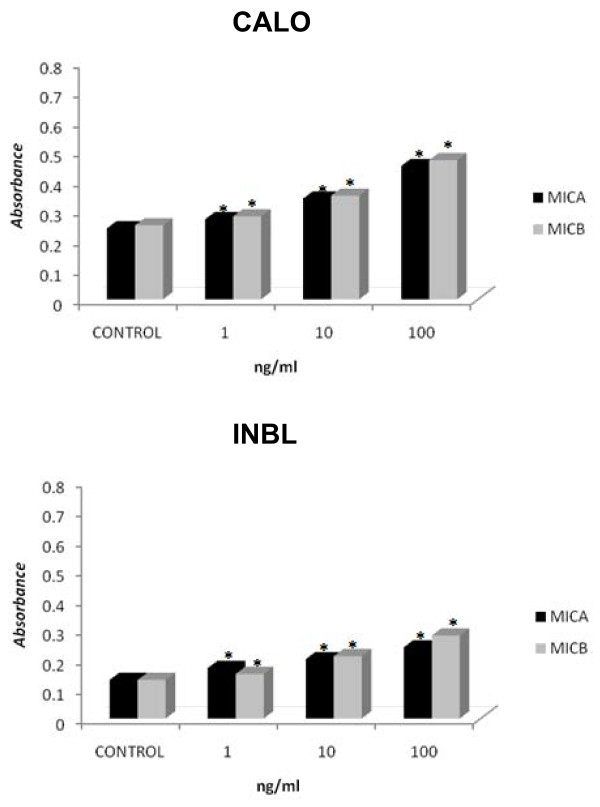
**MICA and MICB induce cervical cancer cell line proliferation**. Cells (5 × 10^3^) were cultured for 72 h in 96-well plates in the presence of 1, 10 or 100 ng recombinant human MICA or MICB. CALO (A) and INBL (B) cell proliferation was then assayed using the MTT technique. * indicates p < 0.05

After we detected the expression of MICA, MICB, and NKG2D in CALO and INBL cells, we proceeded to evaluate if MICA and MICB could modulate their proliferation. For this purpose, we cultured 5 × 10^3 ^CALO and INBL cells for 3 days in the presence of 1, 10, or 100 ng of MICA or MICB and found that both ligands stimulated significant cell proliferation (Figure 7).

## Discussion

The production of MICA and MICB by virus-infection or tumor cells has been previously reported [19,20], and the ability of these ligands to induce cytotoxic activity in NK cells and other cytotoxic lymphocytes through the interaction with their cognate receptor, NKG2D, has been well established [21,22]. Thus, a mechanism by which malignant cells express stress signals, and how other cells recognize those signals to become specifically cytotoxic and mount an immunological response to eradicate the tumor cells, has been clearly established. In this work, we present evidence that both the stress signals and their cognate receptor can be expressed on the same tumor cells. We showed that the leukemic U-937 and TPH-1 myelomonocytic cell lines secrete MICA and MICB, and that those cells also express NKG2D, the receptor for the secreted proteins. We found that ectopic MICA and MICB could induce a strong proliferative response on those cells, suggesting the possibility of an autoregulatory mechanism by which MICA and MICB secreted by the tumor cells are recognized by their own NKG2D receptor to contribute to tumor cell proliferation. The fact that these cells could express and secrete MICA and MICB was expected, because malignant cells are known to express these signal proteins; nevertheless, we were surprised that the same cells expressed NKG2D. We were further surprised when we found that epithelial human cervical cancer cell lines not only expressed MICA and MICB but also their receptor. We do not know why the levels of MICA and MICB took a longer time to be expressed in cervical cells than in myelomonocytic cells but we could speculate that it could be related to their doubling times in vitro because the cervical cells had a doubling time of more than 4 days, while the myelomonocytic ones of less than 3 days. On the other hand we do not know why the myelomonocytic and cervical cells only express membrane NKG2D when they were previously activated by MICA or MICB, but we can speculate that the receptor is mainly expressed intracellularly as suggested in our immunochemistry results and that they were then induced to be expressed on the membrane by MICA and MICB. It is interesting to note that MICA and MICB has a greater induction for proliferation of the myelomonocytic cell lines than in the cervical cancer ones, we think that this is due to the fact that the myelomonocytic cells presented a higher expression of the NKG2D receptor on their membranes.

Our results not only provide evidence that tumor cells can secrete MIC stress molecules and at the same time express their cognate receptor, but demonstrate that non-leukocyte cells, such as epithelial cells, can also express a receptor that was thought to be specific for cytotoxic cells. It would be interesting to determine if this behavior is a more general property of MICA- and MICB-producing cells by evaluating whether virus-infected and tumor cells known to secrete MICA and MICB also express NKG2D. Conversely, it would be interesting to determine if NK and other NKG2D-expressing cells could also be induced to produce and secrete MICA and MICB. If the secretion of MICA and MICB by virus-infected or tumor cells is thought to activate the immunological system through the NKG2D receptor on NK and cytotoxic lymphocytes, then the malignant cells may also present this receptor, as hinted in this work, to help deplete the secreted stress signals in situ and thus avoid activation of the cytotoxic NKG2D-positive cells. This novel idea that tumor cells can express NKG2D could expand a new field of research to discover new mechanisms by which malignant cells escape immunological recognition. We can further speculate that malignant cells not only can deplete MICA and MICB in situ to avoid immune recognition, but they can also use the stress factors as endogenous tumor growth factors. It would be interesting to determine if the simultaneous expression of MICA, MICB and the NKG2D receptor is present in different types of virus-infected and tumor cells. In this respect, the immunosuppressive state that is characteristic of tumor patients and the associated continuous tumor growth warrants further investigation.

## Conclusions

This paper describes two novel findings; one that shows that tumor cells can simultaneously secrete MIC molecules and express their receptor, and another one that tumor epithelial cells (non-leukocytic cells) can also express the NKG2D receptor. The secretion of MIC by tumor cells is thought to activate cytotoxicity through the NKG2D receptor on NK and lymphocytes, then if the malignant cells can also present this receptor as hinted in this work, they could contribute to deplete the secreted stress signals in situ thus avoiding activation of the immunocompetent cells. This novel result that tumor cells can express NKG2D could open a new field of research on new mechanisms by which malignant cells can escape immune recognition.

## Competing interests

The authors declare that they have no competing interests.

## Authors' contributions

BWS and ISC made substantial contributions to conception and design as well as to the interpretation and analysis of the data. CAMC carried out all the experiments reported here. JFMR conceived the study and participated in its design and coordination.

'All authors read and approved the final manuscript'.
